# American Indian/Alaska Native men are less likely to receive prostate-specific antigen testing and digital rectal exams from primary care providers than White men: a secondary analysis of the National Ambulatory Medical Care Survey from 2012–2018

**DOI:** 10.1007/s10552-023-01714-x

**Published:** 2023-05-23

**Authors:** Chris Gillette, Tony Locklear, Ronny Bell, Nathan Bates, Jan Ostermann, Daniel Reuland, Kristie Foley, Cheyenne Lashmit, Sonia Crandall

**Affiliations:** 1 Department of PA Studies, Wake Forest University School of Medicine, Medical Center Blvd., Winston-Salem, NC 27157 USA; 2 Department of Epidemiology and Prevention, Wake Forest University School of Medicine, Winston-Salem, NC USA; 3 Department of Public Health Education, School of Health and Human Sciences, University of North Carolina at Greensboro, Greensboro, NC USA; 4 Pharmaceutical Outcomes and Policy, University of North Carolina Eshelman School of Pharmacy, Chapel Hill, NC USA; 5 Department of Health Services Policy and Management, Arnold School of Public Health, University of South Carolina, Columbia, SC USA; 6 Department of General Medicine, University of North Carolina School of Medicine, Chapel Hill, NC USA; 7 Department of Implementation Science, Wake Forest University School of Medicine, Winston-Salem, NC USA; 8 High Point Family Practice, Atrium Health Wake Forest Baptist, High Point, NC USA

**Keywords:** American Indians or Alaska Natives, Prostatic neoplasms, Early detection of cancer, Health services research

## Abstract

**Purpose:**

(1) Identify the proportion of primary care visits in which American Indian/Alaska Native (AI/AN) men receive a prostate-specific antigen test (PSAT)and/or a digital rectal exam (DRE), (2) describe characteristics of primary care visits in which AI/AN receive PSA and/or DRE, and (3) identify whether AI/AN receive PSA and/or DRE less often than non-Hispanic White (nHW) men.

**Methods:**

This was a secondary analysis of the National Ambulatory Medical Care Survey (NAMCS) during 2013–2016 and 2018 and the NAMCS Community Health Center (CHC) datasets from 2012–2015. Weighted bivariate and multivariable tests analyzed the data to account for the complex survey design.

**Results:**

For AI/AN men, 1.67 per 100 visits (95% CI = 0–4.24) included a PSATs (or PSAT) and 0 visits included a DRE between 2013–2016 and 2018. The rate of PSA for non-AI/AN men was 9.35 per 100 visits (95% CI = 7.78–10.91) and 2.52 per 100 visits (95% CI = 1.61–3.42) for DRE. AI/AN men were significantly less likely to receive a PSA than nHW men (aOR = 0.09, 95% CI = 0.01–0.83). In CHCs, AI/AN men experienced 4.26 PSAT per 100 visits (95% CI = 0.96–7.57) compared to 5.00 PSAT per 100 visits (95% CI = 4.40–5.68) for non-AI/AN men. DRE rates for AI/AN men was 0.63 per 100 visits (95% CI = 0–1.61) compared to 1.05 per 100 (95% CI = 0.74–1.37) for non-AI/AN men. There was not a statistically significant disparity in the CHC data regarding PSA (OR = 0.91, 95% CI = 0.42–1.98) or DRE (OR = 0.75, 95% CI = 0.15–3.74), compared to nHW men.

**Conclusion:**

Efforts are needed to better understand why providers may not use PSA and DRE with AI/AN men compared to nHW men.

**Supplementary Information:**

The online version contains supplementary material available at 10.1007/s10552-023-01714-x.

## Introduction

Approximately 10 million people identify as American Indian/Alaska Native (AI/AN) in the US, comprising about 3% of the total population [1]. Prostate cancer (PC) is the second most common cause of cancer and the second most common cancer-related cause of death in AI/AN men [2, 3]. Relative to non-Hispanic White (nHW) men, AI/AN men have lower incidence of prostate cancer (87.9/100,000 v. 122.2/100,000), but lower survival rates at 1 (98.2 v. 99.1), 5 (95.7 v. 97.9), and 10-year increments (93.4 v. 97.8) [4]. A prior study found that PC in AI/AN men is often discovered at later and more severe stages than non-Hispanic Whites, contributing to this important health disparity [5].

Guidelines for PC screening are heterogeneous and have shifted over the past 20 years, causing significant controversy over when and whom to screen [6]. Most PC is indolent, slow-growing, and will never cause symptoms or reduce length of life. Screening for PC is accomplished using a prostate-specific antigen (PSA) blood test or a digital rectal examination (DRE). Due to the male aging process, older men will have higher PSAs and/or benign prostate nodules compared to younger men [7]. While PC screening tests are generally benign by themselves, a positive test introduces several important risks that could reduce health-related quality of life (e.g., urinary incontinence, impotence) and even mortality [6, 8]. Considering that 75% of men with an elevated PSA and almost 70% of men with prostate nodules do not actually have PC, patients are at a high risk of experiencing complications [9]. The goal, therefore, is to screen the right man at the right time to reduce the risk of complications. Research continues to show that PC screening “reduces [PC] mortality and…metastatic disease” but there is considerable uncertainty regarding the true effect size of PC screening [6]. Due to the underlying uncertainty regarding risks and benefits of PC screening, the only consistency between guidelines is that providers need to use shared decision-making with men so that men can make informed decisions about whether they want to get screened or not.

It is well-known that Black/African American men experience significantly higher rates of PC than other racial/ethnic groups as well as lower relative survival than non-Hispanic White men [4, 10, 11]. While most of the PC disparity literature has centered on Black/African American men, AI/AN men experience similar mortality rates as Black/African American men [4, 12, 13, 14]. In fact, the AI/AN mortality to incidence ratio may be less favorable (0.011) than Black/African American men’s mortality to incidence ratio (0.005), highlighting the importance of studying PC in AI/AN men [4].

Little is known about AI/AN PC screening practices among healthcare providers. Since AI/AN men are accessing care when their PC is more advanced, it is essential to identify how providers are using PSAT and DRE in clinical visits [15, 16]. The Indian Health Service (IHS) only provides care for federally-recognized tribes (about 2.6 million individuals) so it is important to study non-IHS ambulatory health care facilities to obtain a fuller picture of PC screening in AI/AN men [17]. The objectives of this study are to: (1) identify the proportion of visits in which AI/AN men receive a PSA and/or a DRE, (2) describe characteristics of visits in which AI/AN receive PSA and/or DRE, and (3) identify whether AI/AN receive PSA and/or DRE less often than other racial/ethnic groups.

## Methods

This study was approved by the Wake Forest University School of Medicine Institutional Review Board. The Strengthening the Reporting of Observational Studies in Epidemiology (STROBE) statement guided the reporting for this study (Appendix 1) [18]. We conducted a secondary analysis of the National Ambulatory Medical Care Survey (NAMCS) datasets from 2013–2016 and 2018 as well as the NAMCS Community Health Center (CHC) datasets from 2012–2015. The 2017 dataset is not yet available. These datasets were chosen because they are the most recent datasets. We selected multiple years of data due to the changing PC screening recommendations from the United States Preventive Services Task Force (USPSTF) over the years. In 2008, the USPSTF stated that for men younger than 75 years, there was insufficient evidence to assess the benefits and risks of PC screening [19]. In 2012, the USPSTF recommended that providers do not offer PSA-based screening to any man [20]. Then in 2018, the USPSTF recommended that men use shared decision-making when deciding on whether men aged 55–69 years of age should get screened [15]. The NAMCS datasets are publicly available and have been conducted yearly by the National Center for Health Statistics (NCHS) since 1973 [21]. The datasets have been well studied and characterized in previous research. The traditional NAMCS only samples ambulatory, non-federal physician visits while the NAMCS CHC samples ambulatory visits to physicians, physician assistants (PA), and nurse practitioners (NP) at community health centers (e.g., Federally Qualified Health Centers, FQHC). These sites were selected from FQHCs that received Public Health Service Act, Section 330, funding, FQHC look-alikes that do not receive Health Center Program funding, and urban Indian Health Service outpatient clinics [22]. These clinics generally treat medically underserved populations as well as patients who are considered low-income and racial/ethnic minorities, which potentially produce different results than the traditional NAMCS [23]. The NAMCS CHC datasets also provide estimates of how PAs and NPs deliver care whereas the traditional NAMCS only samples physicians. Therefore, we chose to analyze the traditional NAMCS and NAMCS CHC datasets separately.

We included all visits for the traditional NAMCS and the NAMCS CHC dataset in which the visit included: (1) a male, (2), aged 40 years and above, (3) the visit was provided by a General/Family Medicine physician, Internal Medicine physician, physician assistant (PA) or nurse practitioner (NP).

To reduce bias, variable selection was guided by an a priori operationalization of Andersen’s Behavioral Model of Health Services Utilization (ABM) framework (predisposing, enabling, and need constructs) [24]. Next, we accessed the NAMCS documentation to identify and select only those variables, prior to downloading the data. The ABM predisposing factors that we identified from the NAMCS data were: race, ethnicity, and age. The enabling factors that we identified were: type of health insurance (payment), type of physician and/or non-physician clinician that was seen, whether the provider was their primary care provider (PCP), whether the patient was a new patient, and geographic location of the clinic (rural v. non-rural). The need factors that we identified from the dataset were: number of health conditions, number of medications, total number of services provided, and time spent with the provider during the visit. The year of the visit was handled as a categorical variable.

Patient race and ethnicity were measured as categorical variables and age was measured as a continuous variable. Type of health insurance, whether the clinician was the patient’s PCP, and geographic location were measured as categorical variables. Number of health conditions, number of medications, total number of services provided, and time spent during the visit were measured as continuous variables. The NAMCS only samples one clinic visit for each patient and patients are not followed, so all of these variables were measured the day of the visit. For each included year, the NAMCS data were collected using a computer-assisted automated tool that is completed by United States Census Bureau Field Representatives. Data were abstracted from medical charts. More information on how datasets from each year were collected can be found in the NAMCS downloadable documentation [25].

### Statistical analyses

All analyses were conducted using SAS v9.4 (Cary, NC). First, we present descriptive statistics of the visit characteristics using means (with standard deviations) for quantitative variables and proportions (with frequencies) for categorical variables. The dependent variables of interest was whether the visit included a recording of a PSAT (Yes = 1, No = 0) and/or DRE (Yes = 1, No = 0). Next, we compared visit characteristics with AI/AN men versus non-Hispanic White men using a simple survey-weighted logistic regression to account for the complex survey design, treating race as a categorical variable. Each dataset contains a variable (PATWT) that is an inflation factor to produce national estimates. A priori*,* we created multivariable logit models to identify predictors of a PSA using variables that were statistically significant in weighted bivariate statistical testing. We used weighted chi-square tests to test for associations between categorical variables and weighted *t* tests for associations with continuous variables. Variables that were significant in the bivariate testing and that were included in the multivariable model for the traditional NAMCS visits included race, total number of services provided/ordered during the visit, whether a DRE was provided/ordered, whether the clinic was a solo or non-solo clinic, whether the clinic was located in an urban area, and time spent during the visit. Since there were no visits for AI/AN men that included a DRE in the traditional NAMCS dataset, we could not conduct any bivariate or multivariate testing.

For the NAMCS CHC dataset, we conducted weighted bivariate testing comparing PSA and DRE utilization between races. Since there was not a statistically significant relationship with PSA and DRE utilization we did not conduct further testing. Results are presented per 100 visits as well as unadjusted [i.e., crude (cOR)] and adjusted (aOR) odds ratios. All statistical tests were two-sided with an α < 0.05 to indicate statistical significance.

## Results

Table 1 presents visit characteristics for the NAMCS datasets. Across all 5 years of the traditional NAMCS, there were 509,737,580 weighted visits (unweighted = 11,787), of which 232,998 (unweighted = 49) were for AI/AN men. The prevalence rate of PSAs being ordered for AI/AN men was 1.67 per 100 visits (95% CI = 0–4.24, 37,426 weighted visits, unweighted = 2) and 0 visits that included a DRE for AI/AN men. The prevalence of PSAs being ordered for non-AI/AN men was 9.35 per 100 visits (95% CI = 7.78–10.91, 47,428,577 weighted visits, unweighted = 1,025) that included a PSA and the prevalence of DRE was 2.52 per 100 visits (95% CI = 1.61–3.42, 12,767,413 weighted visits, unweighted = 315).

**Table 1:** National ambulatory medical care survey visit characteristics for men aged 40 and over (unweighted n = 11,787; weighted n = 509,737,580)

Visit characteristic	Overall weighted frequency (%, unweighted n)	NA Weighted frequency (%, unweighted n) (n = 2,247,687, unweighted n = 49)	Non-native weighted frequency (%, unweighted n) (n = 507,489,893, unweighted n = 11,738)	*p* value
Survey year				0.01*
2013	105,616,500 (20.72, 4,633)	183,157 (0.17, 17)	105,433,344 (99.83, 4,616)	
2014	102,575,758 (20.12, 4,372)	483,495 (0.47, 15)	102,092,263 (99.53, 4,357)	
2015	111,992,843 (21.97, 1,338)	129,541 (0.11, 5)	111,863,302 (99.88, 1,333)	
2016	90,638,995 (17.78, 848)	1,218,496 (1.34, 11)	89,420,498 (98.66, 837)	
2018	98,913,484 (19.40, 596)	232,998 (0.24, 1)	98,680,486 (99.76, 595)	
Type of payment				N/A
All sources blank	19,210,520 (3.77, 367)	0	19,210,520 (3.79, 367)	
Unknown	15,706,573 (3.08, 536)	53,438 (2.38, 2)	15,653,134 (3.08, 534)	
Private insurance	239,448,311 (46.97, 5,429)	733,247 (32.62, 14)	238,715,064 (47.04, 5,415)	
Medicare	183,908,876 (36.08, 4,339)	500,204 (22.25, 20)	183,408,672 (36.14, 4,319)	
Medicaid	31,072,842 (6.10, 593)	531,805 (23.66, 8)	30,541,037 (6.02, 585)	
Worker's compensation	3,074,570 (0.60, 65)	0	3,074,570 (0.61, 65)	
Self-pay	1,208,517 (2.37, 316)	232,998 (10.37, 1)	11,852,119 (2.34, 315)	
No charge/charity	846,126 (0.17, 9)	0	846,126 (0.17, 9)	
Other	4,384,645 (0.86, 133)	195,994 (8.72, 4)	4,188,651 (0.83, 129)	
Major reason for visit				N/A
Blank	10,238,985 (2.01, 228)	7,325 (0.33, 1)	10,231,660 (2.01, 227)	
New problem	144,818,844 (28.41, 3,708)	769,845 (34.25, 14)	144,048,999 (28.38, 3,694)	
Chronic problem, routine	187,471,786 (36.78, 4,185)	813,726 (36.20, 20)	186,658,060 (36.78, 4,165)	
Chronic problem, flare-up	32,204,398 (6.32, 804)	290,506 (12.92, 5)	31,913,892 (6.29, 799)	
Pre-/post-surgery	10,570,032 (2.07, 214)	0	10,570,032 (2.08, 214)	
Preventive care	124,433,535 (24.41, 2,648)	366,285 (16.30, 9)	124,067,250 (24.45, 2,639)	
Patient's PCP—yes	446,935,103 (87.68, 9,969)	2,007,337 (89.31, 39)	444,927,765 (87.67, 9,930)	N/A
Established patient—yes	470,931,959 (92.39, 10,778)	2,103,819 (93.60, 46)	468,828,141 (92.38, 10,732)	0.82
Type of physician seen—allopathic	451,842,755 (88.64, 10,232)	2,176,093 (96.81, 44)	44,966,663 (88.60, 10,188)	0.03
Non-solo practice—yes	335,287,558 (65.78, 7,926)	1,894,344 (84.28, 43)	333,393,214 (65.69, 7,883)	0.20
Rural clinic—yes	70,040,673 (13.74, 2,017)	401,721 (17.87, 8)	69,638,952 (13.72, 2,009)	0.74
Physician assistant seen during visit—yes	26,588,296 (5.22, 492)	242,991 (10.81, 2)	26,345,305 (5.19, 490)	0.43
Nurse practitioner seen during visit—yes	10,392,870 (2.04, 278)	0	10,392,870 (2.05, 278)	N/A
Patient age, years—mean (SE)	62.19 (0.32)	60.28 (3.14)	62.20 (0.32)	0.54
Total number of chronic conditions—mean (SE)	2.11 (0.06)	3.53 (0.54)	2.10 (0.06)	< 0.01*
Number of medications coded—mean (SE)	4.84 (0.17)	7.95 (0.90)	4.83 (0.17)	< 0.001*
Total number of services ordered/provided including vital signs—mean (SE)	6.81 (0.16)	7.67 (0.88)	6.81 (0.16)	0.33
Time spent with physician (min)—mean (SE)	21.38 (0.31)	25.93 (2.52)	21.36 (0.31)	0.07

*AI/AN* American Indian/Alaska Native, *PCP* primary care physician

*Indicates statistically significant difference at *p* < 0.05

AI/AN men were more likely to have visits with allopathic physicians (χ^2^ = 4.48, DF = 1, *p* = 0.03), had a higher number of chronic conditions (3.53 v. 2.10, *p* < 0.01), and a higher number of medications (7.95 v. 4.83, *p* < 0.001) than non-AI/AN men. There were no other statistically significant visit differences between AI/AN and non-AI/AN men.

AI/AN men were significantly less likely to have a visit that included a PSA than other racial/ethnic groups (cOR = 0.15, 95% CI = 0.03–0.89). PSAs were conducted in 2013 (n = 1) and 2014 (n = 1), both when the visit was for preventive care (n = 2), both when the physician was their PCP (n = 2), both when their provider was an allopathic physician (n = 2), both were in group practice (n = 2), and both were conducted in an urban area (n = 2). Table 2 presents the unadjusted and adjusted multivariable logit models predicting PSA being ordered/provided for men over the age of 40 years in the traditional NAMCS datasets. AI/AN men were significantly less likely to receive a PSA than non-AI/AN men (aOR = 0.09, 95% CI = 0.01–0.83), controlling for number of services ordered/provided, whether a DRE was ordered/provided, whether the clinic was a solo clinic, visit length, year, and whether the clinic was located in a rural area.

**Table 2:** Unadjusted and adjusted survey-weighted odds ratios examining American Indian men’s likelihood of receiving a PSA from primary care providers in Traditional NAMCS

Variable	Weighted n (%, unweighted n)	Unadjusted OR: PSAT	Adjusted OR: PSAT
American Indian/Alaskan native—yes	2,247,687 (0.44, 49)	0.16 (0.03–0.95)	0.09 (0.01–0.83)*
Number of services ordered/provided—mean (SE)	6.81 (0.16)	1.38 (1.32–1.45)	1.37 (1.30–1.44)*
DRE ordered/provided	12,767,413 (2.50, 315)	10.93 (5.41–22.07)	2.21 (1.15–4.24)*
Type of practice—non-solo	335,287,558 (65.78, 7,926)	1.46 (1.02–2.10)	0.93 (0.65–1.33)
Type of practice—rural	70,040,673 (13.74, 2,017)	0.50 (0.33–0.75)	0.88 (0.61–1.28)
Time spent during visit, min—mean (SE)	21.38 (0.31)	1.03 (1.02–1.04)	1.01 (0.99–1.02)

Data are from traditional NAMCS clinic visits

*PSAT* prostate-specific antigen test, *DRE* digital rectal exam

*Statistically significant *p* < 0.05

Table 3 presents visits for the NAMCS CHC datasets. Across 4 years of the CHC datasets, there were 38,452,813 weighted visits (unweighted = 31,955), of which 320,189 (unweighted = 418) were for AI/AN men. The prevalence rate of PSA for all men was 5.03 per 100 visits (95% CI = 4.40–5.67) and the prevalence rate of DRE was 1.05 per 100 visits (95% CI = 0.75–1.37). For non-AI/AN men, there were 1,921,323 weighted visits that included a PSA [unweighted = 1,665, 5.00 per 100 visits (95% CI = 4.40–5.68)] and 402,065 weighted visits [unweighted = 293, 1.05 per 100 (95% CI = 0.74–1.37)] that included a DRE for non-AI/AN men.

**Table 3:** National ambulatory medical care survey-community health center visit characteristics for men aged 40 and over (unweighted n = 31,955, weighted n = 38,452,813)

Visit characteristic	Overall weighted frequency (%, unweighted n)	AI/AN weighted frequency (%, unweighted n) (n = 320,189, n = 418)	Non AI/AN weighted frequency (%, unweighted n) (n = 38,132,624, n = 31,537)	*p* value
Survey year				0.12
2012	11,108,922 (28.89, 6,098)	118,157 (1.06, 145)	10,990,765 (98.94, 5,953)	
2013	8,262,340 (21.49, 9,068)	89,972 (1.09, 94)	8,172,368 (98.91, 8,974)	
2014	9,743,926 (25.34, 9,074)	65,694 (0.67, 118)	9,678,231 (99.33, 8,956)	
2015	9,337,626 (24.28, 7,715)	46,366 (0.50, 61)	9,291,259 (99.50, 7,654)	
Type of payment				
All sources of payment are blank	552,554 (1.44, 395)	1,121 (0.35, 2)	551,432 (1.45, 393)	< 0.001*
Unknown	1,600,432 (4.16, 1,617)	7,618 (2.38, 13)	1,592,814 (4.18, 1,604)	
Private insurance	6,405,520 (16.66, 5,965)	40,658 (12.70, 64)	6,364,862 (16.69, 5,901)	
Medicare	9,650,412 (25.10, 8,467)	73,066 (22.82, 100)	9,577,346 (25.12, 8,367)	
Medicaid or CHIP	11,108,664 (28.89, 8,217)	78,671 (24.57, 111)	11,029,993 (28.93, 8,106)	
Worker’s compensation	96,117 (0.25, 74)	917.84 (0.29, 1)	95,199 (0.25, 73)	
Self-pay	5,253,128 (13.66, 4,960)	46,961 (14.67, 49)	5,206,167 (13.65, 4,911)	
No charge/charity	840,761 (2.19, 667)	6,854 (2.14, 17)	833,907 (2.19, 650)	
Other	2,945,225 (7.66, 1,593)	64,321 (20.08, 61)	2,880,904 (7.55, 1,532)	
Major reason for visit				
Blank	618,053 (1.61, 438)	1,379 (0.43, 5)	616,674 (1.62, 433)	< 0.01*
New problem (less than 3 months onset)	11,732,703 (30.51, 9,859)	139,747 (43.65, 161)	11,592,956 (30.40, 9,698)	
Chronic problem, routine	16,723,429 (43.49, 13,898)	112,927 (35.27, 163)	16,610,502 (43.56, 13,735)	
Chronic problem, flare-up	3,491,064 (9.10, 2,590)	33,016 (10.31, 41)	3,458,048 (9.07, 2,549)	
Pre-/post-surgery	378,642 (0.98, 249)	1,081 (0.34, 2)	377,561 (0.99, 247)	
Preventive care	5,508,922 (14.33, 4,921)	32,039 (10.01, 46)	5,476,882 (14.36, 4,875)	
Patient's PCP—yes	29,320,654 (76.25, 24,265)	185,277 (57.86, 261)	29,135,378 (76.41, 24,004)	< 0.0001*
Established patient—yes	33,854,440 (88.04, 28,105)	291,281 (90.97, 380)	33,563,159 (88.02, 27,725)	0.39
Type of doctor seen				
M.D.—doctor of medicine	19,574,733 (50.91, 15,292)	113,588 (35.48, 157)	19,461,145 (51.04, 15,135)	< 0.001*
D.O.—doctor of osteopathy	2,767,583 (7.20, 2,390)	10,066 (3.14, 13)	2,757,517 (7.23, 2,377)	
Non-physician clinician (PA or NP)	16,110,497 (41.90, 14,273)	196,535 (61.38, 248)	15,913,962 (41.73, 14,025)	
Physician assistant seen during visit—yes	6,404,247 (16.65, 4,842)	70,744 (22.09, 108)	6,333,503 (16.61, 4,734)	
Nurse practitioner seen during visit—yes	9,083,197 (23.62, 8,871)	107,675 (33.63, 110)	8,975,522 (23.54, 8,671)	
Non-solo practice—yes	34,640,408 (90.09, 27,792)	289,559 (90.43, 360)	34,350,849 (90.08, 27,432)	N/A
Rural clinic—yes	8,995,109 (23.39, 9,557)	131,020 (40.92, 207)	8,864,089 (23.25, 9,350)	< 0.01*
Patient age in years	56.53 (0.18)	56.04 (1.07)	56.53 (0.18)	0.65
Total number of chronic conditions	1.96 (0.03)	1.68 (0.12)	1.96 (0.03)	0.02*
Number of medications coded	4.62 (0.09)	4.27 (0.30)	4.62 (0.09)	0.26
Total number of services ordered or provided including vital signs	7.08 (0.09)	6.57 (0.39)	7.08 (0.09)	0.20
Time spent with physician in minutes	19.95 (0.27)	22.50 (1.21)	19.93 (0.27)	0.03*

Data are from traditional NAMCS clinic visits

*SE* standard error, *PA* physician assistant, *NP* nurse practitioner

*Statistically significant *p* < 0.05

In contrast, there were 13,651 weighted visits [unweighted = 19, 4.26 per 100 visits (95% CI = 0.96–7.57)] that included a PSA and 2015 weighted visits [unweighted = 3, 0.63 per 100 visits (95% CI = 0–1.61)] that included a DRE for AI/AN men. AI/AN men were significantly more likely to: (a) have a visit with a PA or NP (χ^2^ = 13.01, *p* < 0.001), (b) have the provider record the major reason for the visit as a new problem compared to preventive care (OR = 2.06, 95% CI = 1.21–3.52), (c) had ‘Other’ type of insurance compared to private insurance (OR = 3.50, 95% CI = 1.35–9.02), (d) had fewer chronic conditions (1.68 v. 1.96, *p* = 0.02), and (e) had longer visits (22.50 min v. 19.93 min, *p* = 0.03) than non-AI/AN men in the CHC dataset.

There was not a statistically significant relationship between a PSA being ordered/provided and whether the patient was a AI/AN man (cOR = 0.91, 95% CI = 0.42–1.98) or for DREs (cOR = 0.75, 95% CI = 0.1–3.74), compared to non-Hispanic White men in the CHC dataset. No further multivariable inferential statistical testing was conducted due to there not being a statistically significant finding from bivariate testing.

## Discussion

We found evidence that AI/AN men experience a significant health care disparity that could potentially impact PC mortality. At the national level, AI/AN men were significantly less likely to receive a PSA and DRE than non-AI/AN men during the years 2013–2016 and 2018 during physician visits to non-federal clinics. What is particularly concerning is that there were zero instances of DREs in the traditional NAMCS over the entire five-year period and there were no PSAs conducted after 2014.

This is an important finding because data are clear that AI/AN men experience disproportionately greater PC mortality compared to other racial/ethnic identities [4]. Given that AI/AN men were less likely to receive a PSA and that no AI/AN men received a DRE, it is necessary to better understand why healthcare providers are not using PSA and DRE in AI/AN men compared to other racial/ethnic identities. We also found that this disparity may not exist when AI/AN men visit CHCs. More research is needed to better understand this potential disparity in access to important services.

Our results are consistent with CDC data as well as prior research showing that AI/AN men are not necessarily predisposed to poor PC outcomes, but that they present for care when their PC is more advanced compared to other racial/ethnic groups [5]. The American Urological Association and USPSTF PC screening guidelines recommend that providers use shared decision-making to discuss the benefits and risks of PSA and DRE. Due to the way the data in our study are collected, it is impossible to know whether shared decision-making was used when discussing PSA and/or DRE and that AI/AN men chose not to receive a PSA and/or DRE during their visit [15, 16]. Future research should be conducted to examine how providers discuss PSA and DRE with their AI/AN patients as well as whether subsequent decisions are consistent with AI/AN men’s values and preferences.

Prior research in Black/African American men also found disparities in access to PSA and DRE, which led to guidelines encouraging the use of PSA and DRE in younger Black/African American men [12, 13]. Based on the fact that AI/AN men in the 50–59 year age range are more likely to be diagnosed with PC, it may be time the guidelines also specifically encourage providers to screen AI/AN men for PC at younger ages, similar to past actions for Black/African American men [26]. Future research also needs to identify whether providers experience any difficulties regarding PSA and DRE for AI/AN men, such as lack of insurance or lack of continuity of care [27, 28, 29].

The PC screening guidelines are heterogeneous among different organizations, which may cause confusion among providers [30]. Due to the fact that most PC is indolent and benign and that the downstream effects of screening introduces the risk of significant complications, most research has focused on trying to identify the right person to screen at the right time with a goal to reduce false positives. Making the decision to screen a man for PC is a difficult decision that has pros and cons regardless of the guidelines, which is why using shared decision-making is so important [15]. In 2012, the USPSTF recommended that providers not offer PSA-based PC screening. Recent research has called the 2012 recommendation into question because prior to the 2012 USPSTF recommendations, new metastatic PC cases and PC mortality had been decreasing [31]. After the 2012 recommendations, there has been a steady increase in metastatic PC cases, potentially because providers have reduced their PC screening behaviors [31, 32]. In 2018, the USPSTF changed their recommendation from a Grade D (discourage the service) to a Grade C (offer the service to select patients) for men aged 55–69 years of age after using shared decision-making.

While the results of our study are novel and important, there are limitations that affect generalizability of findings. First, the case counts for PSA and DRE in AI/AN men are below the National Center for Health Statistics’ guidelines for stability of findings (n = 30), therefore, our findings about statistical significance should be interpreted with caution [33]. Second, we included all visits (pre- and post-surgery) and all payer types (e.g., Worker’s Compensation visit), which may inflate the denominator for the visits in which PSA and DRE would not be ordered/provided. Reasons for visits are often multifaceted in middle-aged and older adults, therefore, we chose to include all types of visits and payers. Also, these data reflect AI/AN men who do not access IHS facilities for care and our results may not be generalizable to IHS visits. The IHS provides access to health care for approximately one-fourth of all AI/AN and is only provided to members of federally-recognized tribes, so it is important to better understand these populations’ care patterns. We used the unimputed race variable to identify AI/AN men and there are known limitations when that variable is used instead of the imputed race variable, which does not include AI/AN status, which may contribute to a Type II error in our data.

While our study, like others, has limitations, our results shine a light on a potential difference in screening practices for AI/AN men compared to non-Hispanic White men in the US. Visits for AI/AN men were found to be less likely to include a PSA and DRE compared to other race/ethnic identities, even after adjusting for visit and provider characteristics. Future research needs to identify methods to improve the uptake of PSA and DRE in AI/AN men to reduce the likelihood of AI/AN men presenting for care when their PC is more advanced and more aggressive compared to other racial/ethnic identities.

## Supplementary Information

Below is the link to the electronic supplementary material.

Supplementary file1 (DOCX 29 KB)

## Data Availability

Datasets are publicly available.
